# A systematic CRISPR screen reveals an NBL1-mediated Jak/Stat3 crosstalk to promote ovarian cancer metastasis

**DOI:** 10.1016/j.gendis.2025.101740

**Published:** 2025-06-28

**Authors:** Yue Qi, Wenwen Zhang, Xinyu Li, Yi Shi, Pengpeng Qu

**Affiliations:** a Clinical School of Obstetrics and Gynecology Center, Tianjin Medical University, Tianjin 300100, China; b Department of Gynecological Oncology, Tianjin Central Hospital of Gynecology Obstetrics, Tianjin 300100, China; c The School of Medicine, Nankai University, Tianjin 300071, China

**Keywords:** CRISPR/Cas9 screen, EMT, Jak/stat3, *NBL1*, Ovarian cancer metastasis

## Abstract

Patients with ovarian cancer (OC) are at high risk of developing transcoelomic metastasis in the early stages, which is strongly associated with increased mortality rates. However, the mechanism by which OC cells disseminate from the primary site and colonize distant sites remains unknown. Here, through an *in vivo* genome-wide CRISPR/Cas9 screen, we identified NBL1, which increased dramatically in OC patients during peritoneal metastasis, as a key factor promoting the transcoelomic metastasis of OC. Overexpression of NBL1 in OC cells greatly promotes the transcoelomic metastasis. When OC cells disseminate into the peritoneal cavity, they induce the transition of peritoneal epithelial cells to mesothelial cells, ultimately activating the Jak/Stat3 signaling pathway. Thus, we show a NBL1-mediated crosstalk between peritoneum epithelial cells and mesothelial cells that supports a metastasis-promoting process.

## Introduction

The incidence of ovarian cancer (OC) ranks third among all gynecologic malignancies, but OC has the highest mortality, which threatens women's health worldwide seriously.^1^ According to the global cancer statistics (GLOBOCAN 2020) released by the International Agency for Research on Cancer (IARC), there were about 314,000 new cases and 207,000 deaths of OC in 2020.^2^ Due to the lack of specific symptoms in the early stage, 70% of patients are diagnosed as advanced cases (stages III or IV),^3^ and the 5-year survival rate is about 40%.^4^ Besides, most of the recurrence and death of OC patients are closely related to metastasis, which contributes to the poor clinical outcomes.^5^ Therefore, the mechanism of metastasis and recurrence needs to be profoundly elucidated.

OC could metastasize to distant organs by blood and lymph nodes, with transcoelomic metastasis being most common.^6^ This complex process involves detachment, immune evasion, and implantation,^7^ with epithelial to mesenchymal transition (EMT) playing an essential role,^8^ driven by cytokines and hormones^9^, ^10^, ^11^ that promote OC metastasis.^12^ Janus kinase/signal transducers and activators of transcription (JAK-STAT) is a conserved signaling pathway in animals that transmits extracellular signals to DNA, affecting transcription and cellular processes like proliferation and apoptosis.^13^ Therefore, it is imperative to understand the specific mechanism in OC for developing more efficient strategies against metastatic OC.

To identify the key in peritoneal metastasis of OC, we performed CRISPR/Cas9 genome-wide knockout screening in an orthotopic mouse model. Neuroblastoma suppressor of tumorigenicity 1 (NBL1), also known as DAN, has been widely reported as a tumor suppressor in multiple cancers, including pancreatic cancer and lung adenocarcinoma.^14^ Intriguingly, our CRISPR screening and clinical data revealed a paradoxical role of NBL1 in OC, namely, NBL1 is significantly up-regulated in metastatic lesions and correlates with poor prognosis, suggesting a metastasis-promoting function. This finding challenges the conventional view of NBL1 as a tumor suppressor and highlights its context-dependent roles in cancer progression. Here, we aimed to dissect the molecular mechanisms underlying this dual role, with a focus on its interaction with the Jak/Stat3 pathway.

## Materials and methods

### Cell culture

A2780, SK-OV-3, 3AO, and HEK 293T cells were purchased from the American Type Culture Collection. ID8 cells were purchased from Merck and screened in C57BL/6 mice for highly metastatic cells. SK-OV-3 cells were cultured in McCoy's 5A medium. A2780 and 3AO cells were cultured in RPMI 1640 medium. HEK 293T and ID8 cells were cultured in Dulbecco's modified Eagle's medium. All the mediums were supplemented with 10% fetal bovine serum (BI, China) and 1% penicillin–streptomycin (Invitrogen, China) and maintained in an atmosphere of 5% CO_2_ and 95% air at 37 °C.

### CRISPR/Cas9 knockout *in vivo* screening

The method for the highly metastatic gene screening process using the CRISPR/Cas9 library has been established.^15^, ^16^ Briefly, the procedure was as follows: the GeCKO v2 human sgRNA library (developed by Zhangfeng's lab and obtained from Addgene) contains 122,756 sgRNAs targeting 19,050 protein-coding genes and 1864 miRNAs, with an average of 6 sgRNAs per gene to ensure coverage redundancy. SK-OV-3 cells were transduced with the library at a multiplicity of infection (MOI) of 0.5, followed by puromycin selection (2 μg/mL, 7 days) to achieve >90% infection efficiency. Cells (2 × 10^6^) were orthotopically injected into 8-week-old female NOD-SCID mice (*n* = 10 per group). After 40 days, metastatic nodules were isolated and expanded *in vitro*. Three iterative cycles of *in vivo* selection were performed to enrich metastasis-promoting sgRNAs. High-throughput sequencing data were analyzed using RIGER P, with sgRNAs retained if they met |log_2_fold-change| ≥1 and adjusted *p*-value < 0.05. To minimize off-target effects, we validated the top 100 candidate genes using 3 independent sgRNAs per gene in secondary functional assays.

### Animal models and experiments

NOD-SCID mice (immune-deficient, strain code 001303) were selected for SK-OV-3 (human ovarian cancer) xenografts to avoid graft rejection, while C57BL/6 mice (immune-competent, strain code 000664) were used for ID8 (murine ovarian cancer) orthotopic models to mimic syngeneic tumor–microenvironment interactions. Mice were randomized into groups post-injection, and investigators were blinded to group assignments during data collection. Animal experiments were approved by the Ethics Committee of Nankai University. All animals were housed and maintained in the Nankai University Animal Facility.

### Cell viability assay

Cell viability was assessed using the cell counting-8 kit (CCK-8, Beyotime, China) according to the manufacturer's instructions. 3AO, A2780, and ID8 cells were seeded in 96-well plates at a density of 5 × 10^3^ cells per well and cultured for specific periods, followed by the addition of 10 μL of CCK8 reagent and incubated at 37 °C for 2 h. The optical density value was then measured at a wavelength of 450 nm (Thermo Fisher Scientific).

### Wound-healing assay

Cells were cultured in a 6-well plate until reaching 80%–100% confluence, and then scratched with a sterilized 10 μL pipette tip. The cells were subsequently washed with 1 × phosphate-buffered saline (PBS) solution to remove any suspended cells. Following this, the different groups of conditioned media were replaced and cultured for 24 h and 48 h in a 37 °C incubator. Images were captured using microscopy at the same point of the wound marked on the plate.

### Colony formation assay

A total of 1000 cells were seeded into 6-well plate and cultured in a medium containing 10% fetal bovine serum for 2 weeks. Afterward, the colonies were washed with PBS solution, fixed in 4% paraformaldehyde for 15 min, and stained with crystal violet solution for 30 min. The numbers of colonies were then quantified using ImageJ software.

### Transwell assay

Transwell assays were utilized to assess the invasive capabilities of the cells. For the invasion assay, transwell chambers were coated with Matrigel (Corning) at 37 °C for 2 h. The cells (5 × 10^4^) were suspended in 200 μL of serum-free medium and seeded into the upper chamber. Subsequently, 650 μL of 10% fetal bovine serum medium was added to the lower chamber. Following a 48-h incubation period, the invasive ability was evaluated as previously described for the colony formation assay.

### RNA extraction and real-time quantitative PCR

According to the manufacturer's instructions, total RNA was extracted with TRIzol (Invitrogen, Thermo Scientific, USA), quantified via NanoDrop, reverse-transcribed using a cDNA reverse transcription kit (Mei5bio, Beijing, China), and amplified through PCR at 95 °C for 30 s, followed by 40 cycles of 95 °C for 10 s and 60 °C for 34 s in a 7500 real-time PCR system with SYBR green (Bimake, Shanghai, China). GAPDH mRNA expression served as the internal control. Relative mRNA expression levels were determined using the 2^−ΔΔCt^ method. All primers used for real-time quantitative PCR were synthesized by Jin Weizhi (Tianjin, China) and are listed as follows: NBL1-Human: forward primer: TTGGTGAGGTCTGCAGTGGAAC, reverse primer: ACCAGGACCCGAAGCATCAT; NBL1-mice: forward primer: CCCGCCACCTATCAACAAG, reverse primer: CCCACAATCTGCGTGATGTTC; Gadph: forward primer: AGAAGGCTGGGGCTCATTTG, reverse primer: AGGGGCCATCCACAGTCTTC.

### Protein extraction and western blotting

Western blot analyses were independently repeated three times. Proteins were extracted using RIPA buffer (Solarbio) and quantified via the bicinchoninic acid assay (Thermo Fisher). For each experiment, 40–50 μg of protein per sample was subjected to SDS-PAGE and then transferred to PVDF membranes (Millipore). Membranes were probed with primary antibodies at 4 °C overnight, followed by horseradish peroxidase-conjugated secondary antibodies. Blots were developed using enhanced chemiluminescence detection reagent, and band intensities were quantified with ImageJ (normalized to GAPDH or α-Tubulin).

### RNA-sequencing and bioinformatics analysis

Total RNA was extracted after NBL1 overexpression using TRIzol reagent. RNA-sequencing analysis was performed by an Illumina NovaseqTM 6000 (LC Sciences, Hangzhou, China). Differential gene expression analysis of mRNA was conducted with the R package edgeR or DESeq2. |log_2_fold-change| ≥1 and adjusted *p*-values < 0.05 were set as the criteria for selecting differentially expressed genes between the NBL1 overexpression and control groups. Gene Oncology (GO) analyses of the differentially expressed genes were conducted according to the GO database. Pathway enrichment analyses of the differentially expressed genes were performed referring to the Kyoto Encyclopedia of Genes and Genomes (KEGG) pathway database.

### Establishment of a stable NBL1-overexpression or NBL1-knockdown cell line

ID8 cells with stable NBL1 overexpression or A2780 and 3AO cells with stable NBL1 knockdown were constructed using a lentivirus system (GENECHEM, Shanghai, China). After seeding the cells in 6-well plates at a density of 2 × 10^4^ cells/well, they were incubated overnight with a medium containing lentivirus and HitransG/A, followed by selection with puromycin.

### Annexin V assay

Apoptosis was analyzed by annexin V staining and fluorescence-activated cell sorting (FACS) analysis using an Annexin V-FITC Staining Kit (BioLegend, San Diego, California, USA). The cells were harvested by centrifugation at 1000 *g* for 5 min and washed twice with cold PBS solution. The cells were then resuspended in 1 × binding buffer and stained with annexin V-FITC and propidium iodide according to the manufacturer's instructions. The reaction product was analyzed on a FACS Calibur flow cytometer, and the data were analyzed with FlowJo software (Ver. 10.1, FlowJo, LLC).

### Human tissue specimens

According to cases with the diagnosis of OC, a retrospective collection of 20 OC specimens with paired primary and metastasis tissues was conducted at Tianjin Central Hospital of Gynecology Obstetrics, Tianjin Medical University. These samples were immediately placed on ice upon collection and then frozen and stored at −80 ℃ for long-term preservation. Informed consent was obtained from all patients or their family members. This study complied with the requirements of the Ethics Committee of Tianjin Central Hospital of Gynecology Obstetrics.

### Bioinformatics analysis of clinical data

Raw gene expression data for OC (GSE137237) were retrieved from the Gene Expression Omnibus (GEO) database. Correlation analyses of gene expression data and survival analysis from The Cancer Genome Atlas (TCGA) OC cohort were conducted using the GEPIA analysis tool (http://gepia.cancer-pku.cn/). The Kaplan–Meier Plotter online analysis tool (https://kmplot.com/analysis/) was used to perform Kaplan–Meier analyses of patients with OC.

### Gene expression analysis of NBL1 in pan-cancer

Using the TIMER2.0 database, the differential gene expression between tumor and normal tissues was investigated. In addition, the databases TCGA and Genotype-Tissue Expression (GTEx) were used to investigate several malignancies that lacked normal tissues in TCGA. In our research, a total of 33 tumor expression profiles were demonstrated.

### Gene mutation analysis of NBL1

cBioPortal (https://www.cbioportal.org/) was used to analyze the NBL1 tumor genomic characteristics, including mutation, amplification, and profound deletion. In addition, the clinical prognosis between the modified and unmodified groups was also examined.

### Relationship between NBL1 expression and immune cells infiltration

The relationship between NBL1 expression and immune cells, including CD8^+^ T cells, CD4^+^ T cells, B cells, and tumor-associated fibroblasts, was investigated using TIMER2.0. Positive correlation was indicated by Spearman's > 0 and *p* < 0.05. A negative correlation was indicated by Spearman's < 0 and *p* < 0.05.

### Statistical analysis

Statistical analyses were performed using SPSS 22.0. For comparisons between two groups, two-tailed student's *t*-tests were applied. Multiple groups were analyzed by one-way ANOVA with Bonferroni correction for post-hoc comparisons. Data were presented as mean ± standard error of the mean unless stated otherwise. In figures, error bars represent standard error of the mean, and significance thresholds were set as ∗*p* < 0.05, ∗∗*p* < 0.01, and ∗∗∗*p <* 0.001 after correction.

## Results

### NBL1 is a pro-transcoelomic metastasis gene in OC identified by a genome-scale screening *in vivo*

**Figure 1:**
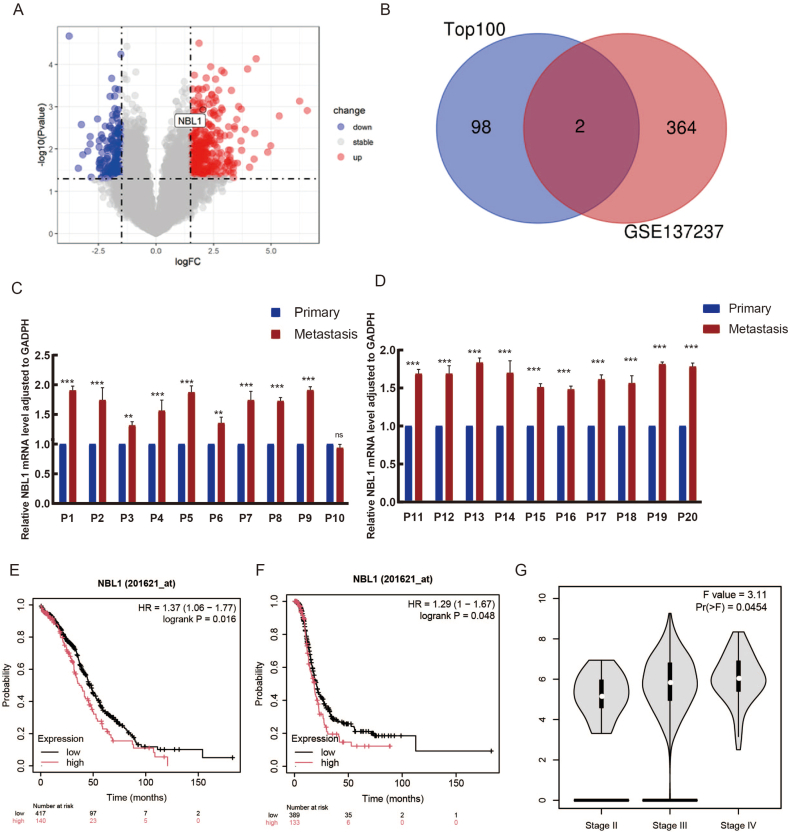
The NBL1 gene plays a crucial role in the metastasis of OC and is associated with a poor prognosis for patients. **(A)** Differences in gene expression are depicted in the figure, with red representing the relatively higher gene expression level and blue representing the relatively lower gene expression. The horizontal dotted line above indicates genes with *P*_adj_ < 0.05, while the vertical dashed lines on both sides represent genes with |log_2_fold-change| > 1.5. **(B)** The Venn diagram compares the hits that meet the specified criteria. **(C**, **D)** Real-time quantitative PCR analysis of NBL1 expression in human primary OC tissues (Primary) and paired peritoneal metastases (Metastasis). Data were normalized to GAPDH (endogenous control) using the 2^−ΔΔCt^ method. Values represent mean ± standard error of the mean from three independent experiments (∗*p <* 0.05, ∗∗*p <* 0.01, ∗∗∗*p <* 0.001; paired two-sided student's *t*-test). **(E**, **F)** Kaplan–Meier survival plot of the overall survival (OS) and progression-free survival (PFS) of serous OC patients with different NBL1 expression. **(G)** Correlation analysis of NBL1 expression with the clinical stage of OC patients. NBL1, neuroblastoma suppressor of tumorigenicity 1; OC, ovarian cancer.

To identify key genes regulating transcoelomic metastasis of OC, we established an orthotopic transplant tumor model using SK-OV-3 cells in NOD-SCID mice and GeCKO v2.0 CRISPR knockout library for whole genome gene knockout.^17^ Through high-throughput DNA sequencing screening and analysis of peritoneal highly metastatic cancer cells, we identified a set of key genes influencing OC metastasis.^15^, ^16^ To further screen for genes highly associated with OC metastasis, we reanalyzed this dataset and utilized the transcriptome sequencing dataset GSE137237^18^ from the public GEO database, which consisted of 11 paired RNA sequencing datasets of OC *in situ* and metastatic tumor tissues. There were 573 differentially expressed genes, including 366 up-regulated genes and 207 down-regulated genes (Fig. 1A). The overlap between the top 100 metastatic genes identified through CRISPR screening and the 366 up-regulated genes identified through GEO analysis revealed the presence of two key metastasis-promoting genes, NBL1 and nucleotide-binding oligomerization domain 1 (NOD1) (Fig. 1B). Notably, NBL1 expression level was significantly higher in metastatic tumors than in primary tumors.

### The NBL1 expression is dramatically increased in metastases of OC patients and positively correlates with the clinical outcomes

To gather clinical evidence on NBL1's relevance in OC metastasis, we analyzed primary OC tissues and paired metastaic tissues from 20 serous OC patients. The expression of NBL1 revealed a significant increase in the transcoelomic spread cancer samples compared with those from the primary sites (Fig. 1C, D). High levels of NBL1 were linked to poorer overall survival and progression-free survival in serous OC patients (Fig. 1E, F) and positively associated with clinical stage (Fig. 1G).

A prognostic multivariate Cox regression analysis was conducted using TCGA data to examine the relationship between NBL1 expression and clinicopathological characteristics in OC (Table S1). Based on the high and low relative expression levels, 381 patients were divided into two groups. NBL1 was significantly overexpressed in advanced stage patients, particularly in stage IV disease (*p* = 0.005), and associated with factors such as venous invasion (*p* = 0.008), tumor status (*p* = 0.002), residual tumor (*p <* 0.001), and overall survival (*p* = 0.024), while showing no significant correlation with initial treatment outcome (*p* = 0.658), age > 60 years (*p* = 0.873), histological grade (*p* = 0.478), lymphatic vessel invasion (*p* = 0.137), or bilateral primary tumors (*p* = 0.982). In conclusion, NBL1 may promote OC progression through various mechanisms.

### Pan-cancer analysis of NBL1 across various cancer types

To investigate the biological role of NBL1 in various cancers, we analyzed its expression profiles using the TIMER2.0 online database, finding high expression in 5 cancer types and low in 11 compared with normal tissues (Fig. S1A). We also examined NBL1 mutations, amplification mutations prevalent in OC, and rare deletions under 1% (Fig. S1B). Deletion mutations are more common in other cancers. Figure S1C illustrates that NBL1 gene mutations in the TCGA database include protein truncating, missense, and splice variants, with a proline (P)-to-arginine (R) conversion observed in NBL1's structure, alongside 12 missense and 1 splice mutation potentially affecting NBL1 expression in OC, leading to significantly lower prognosis for those with NBL1 mutations (Fig. S1D, S1E).

### NBL1 expression is closely associated with immune cell infiltration in various types of cancer

Recent studies highlight the critical role of immune cells, especially T cells and B cells, in the progression of cancer.^19^^,^^20^ Correlation analysis using the TIMER2.0 database revealed that NBL1 expression was negatively correlated with native T cell subsets and B cell numbers in OC (Fig. S2A–S2C), suggesting that high NBL1 level may hinder their immune functions. Additionally, NBL1 showed a positive correlation with cancer-associated fibroblasts across various cancers, including OC (Fig. S2D).

### NBL1 promotes OC cell proliferation, invasion, and migration *in vitro*

**Figure 2:**
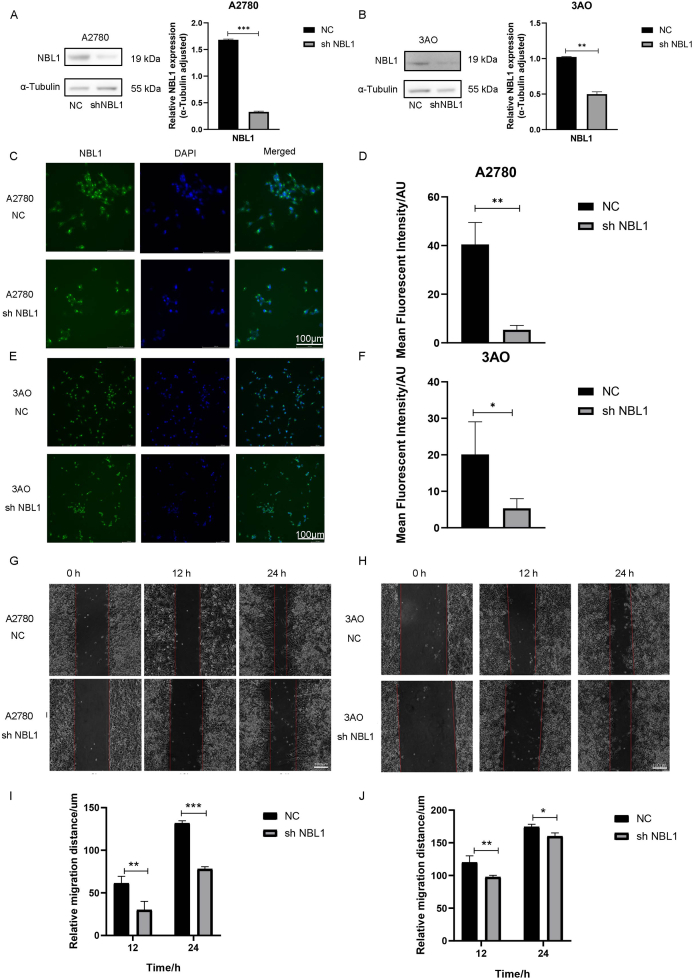
NBL1 down-regulation suppresses the proliferation and migration of ovarian cancer cells. **(A, B)** The validation of the knockdown efficacy of NBL1 in A2780 and 3AO cells by western blotting. **(C–F)** Quantitative analysis of NBL1 using immunofluorescence in A2780 and 3AO cells. **(G**–**J)** Wound-healing assay showed the horizontal migration ability with NBL1 knockdown in A2780 and 3AO cells. Data were shown as mean ± standard deviation. ∗*p* < 0.05, ∗∗*p* < 0.01, and ∗∗∗*p* < 0.001. NBL1, neuroblastoma suppressor of tumorigenicity 1.

To investigate NBL1's role in OC progression, sh-NBL1 lentivirus was employed to knock down NBL1 expression in A2780 and 3AO cells. The knockdown efficiency was assessed by western blotting (Fig. 2A, B) and by measuring the fluorescence signal intensity (Fig. 2C–F). The wound-healing assay showed that reduced NBL1 significantly hindered OC cell migration (Fig. 2G–J).

**Figure 3:**
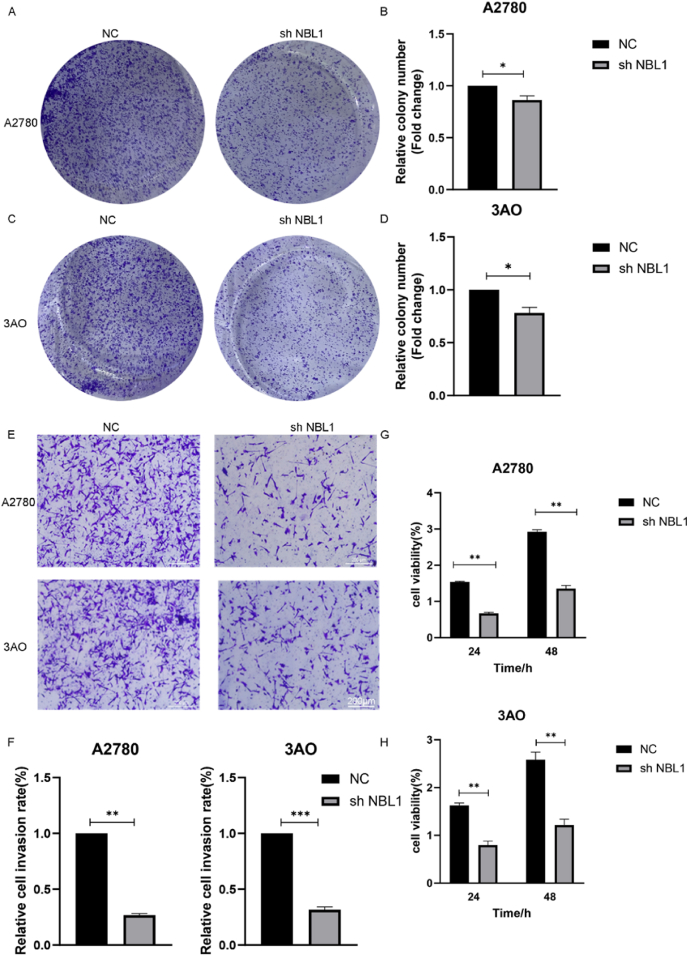
NBL1 down-regulation suppresses the proliferation and invasion of ovarian cancer cells. **(A**–**D)** Effects of NBL1 knockdown on colony formation in A2780 and 3AO cells. **(E, F)** Effects of NBL1 knockdown on invasion in A2780 and 3AO cells. **(G, H)** CCK-8 assay showed the effect of NBL1 knockdown on proliferation in A2780 and 3AO cells. The *y*-axis represents OD450 values measured at 24 h and 48 h. Data were shown as mean ± standard deviation; ∗*p <* 0.05, ∗∗*p <* 0.01, ∗∗∗*p <* 0.001. NBL1, neuroblastoma suppressor of tumorigenicity 1.

Colony formation showed that NBL1 knockdown significantly reduced the proliferation of A2780 and 3AO cells (Fig. 3A–D). Transwell assay indicated that lower NBL1 expression substantially diminished the invasive capability of OC cells (Fig. 3E, F). CCK8 assays confirmed that reduced NBL1 expression inhibited OC cell line proliferation (Fig. 3G, H).

**Figure 4:**
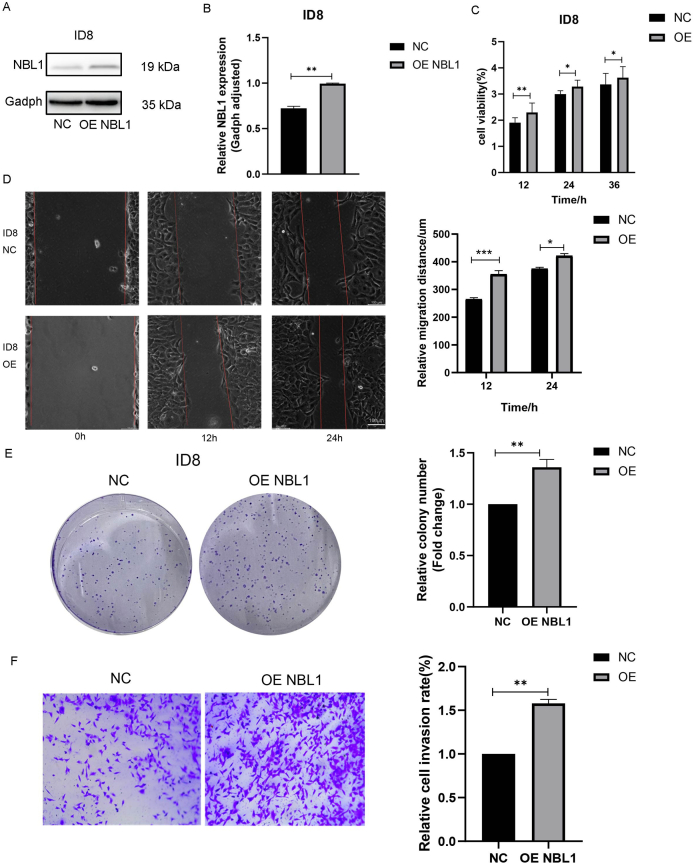
NBL1 promotes ovarian cancer cell proliferation, migration, and invasion *in vitro*. **(A, B)** The validation of the overexpression efficacy of NBL1 in ID8 cells by real-time quantitative PCR and western blotting. **(C)** CCK-8 assay showed the effect of NBL1 overexpression on proliferation in ID8 cells. **(D)** Wound-healing assay showed the horizontal migration ability with NBL1 overexpression in ID8 cells. **(E)** Effects of NBL1 overexpression on colony formation in ID8 cells. **(F)** Effects of NBL1 overexpression on invasion in ID8 cells. Data were shown as mean ± standard deviation. ∗*p* < 0.05, ∗∗*p* < 0.01, ∗∗∗*p* < 0.001. NBL1, neuroblastoma suppressor of tumorigenicity 1.

Our data establish NBL1 as a critical driver of OC metastasis through dual mechanisms: i) direct activation of Jak/Stat3 signaling via physical interaction, and ii) suppression of anti-tumor immunity by limiting T cell infiltration. To this end, we established a mouse OC cell line, ID8, with stably elevated levels of NBL1 (Fig. 4A, B). The overexpression of NBL1 (OE NBL1) enhanced proliferation (Fig. 4C), migration (Fig. 4D), and invasion (Fig. 4F), as shown by increased colony formation (Fig. 4E) and migratory ability (Fig. 4D–F). Collectively, these results highlighted NBL1's crucial role in regulating these processes.

### Transcriptome analysis reveals the potential signal pathways by NBL1 overexpression

To investigate biological behaviors from NBL1 overexpression, OE NBL1 and vector ID8 cells underwent RNA sequencing. 22 up-regulated and 184 down-regulated genes were identified by differentially expressed gene analysis of OE NBL1 cells compared with control cells (Fig. S3A, S3B), along with 93 alternative 3′ splice sites (A3SS) and 85 alternative 5′ splice sites (A5SS) (Fig. S3C) due to splicing alterations.

**Figure 5:**
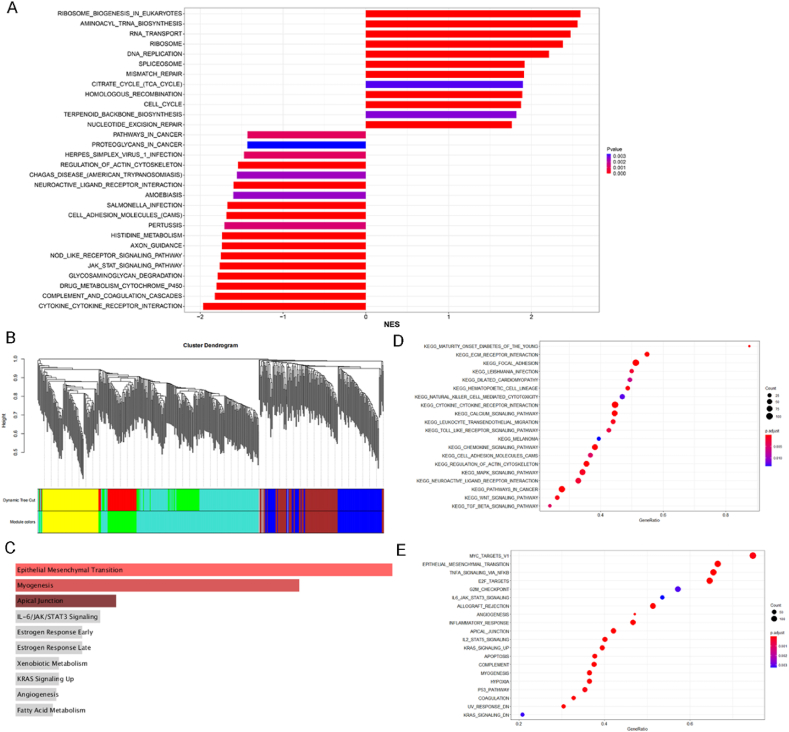
Potential signal pathway analysis by RNA sequencing and online databases. **(A)** Summary plot of the top 30 results of Gene Set Enrichment Analysis (GSEA). **(B)** The analysis involves constructing a weighted gene co-expression network and performing cluster analysis. **(C)** The differential genes in the NBL1 module were subjected to the Kyoto Encyclopedia of Genes and Genomes (KEGG) enrichment analysis. **(D)** The bubble plot for Gene Ontology (GO) enrichment analysis. **(E)** The bubble map for KEGG enrichment analysis. NBL1, neuroblastoma suppressor of tumorigenicity 1.

The mechanism by which NBL1 affects the progression of OC was further elucidated through pathway enrichment analysis. The top 30 positively enriched results revealed significant enrichment in key biological processes such as DNA replication, ribosome biosynthesis, RNA transport, and homologous recombination repair (Fig. S3D). Conversely, among the top 30 negatively enriched results, processes about immune response, cytokine receptor-mediated signaling pathway, cytotoxic response, and iron ion channel activity were enriched (Fig. S3E). With rankings based on the normalized enrichment score (NES), the impact of NBL1 overexpression on cell cycle regulation and Jak/Stat signaling pathways was identified (Fig. 5A).

We analyzed the GSE137237 dataset and sequencing data to study NBL1-mediated OC transfer by weighted gene co-expression network analysis (WGCNA), revealing six gene modules through hierarchical clustering (Fig. 5B). KEGG enrichment analysis of the turquoise-green module showed key pathways such as EMT, myogenesis, and apical junctions (Fig. 5C). Furthermore, significant enrichment of the interleukin-6 (IL-6)/JAK/STAT3 signaling pathway was observed. We reclassified OC patients within the TCGA database based on NBL1 expression, and Gene Set Enrichment Analysis (GSEA) indicated enrichment in GO categories for EMT and apoptosis (Fig. 5D), alongside pathways like IL-6/STAT3 pathway (Fig. 5E).

### NBL1 enhances the resistance to apoptosis, triggers EMT, and activates the Jak/Stat3 signaling pathway in OC

**Figure 6:**
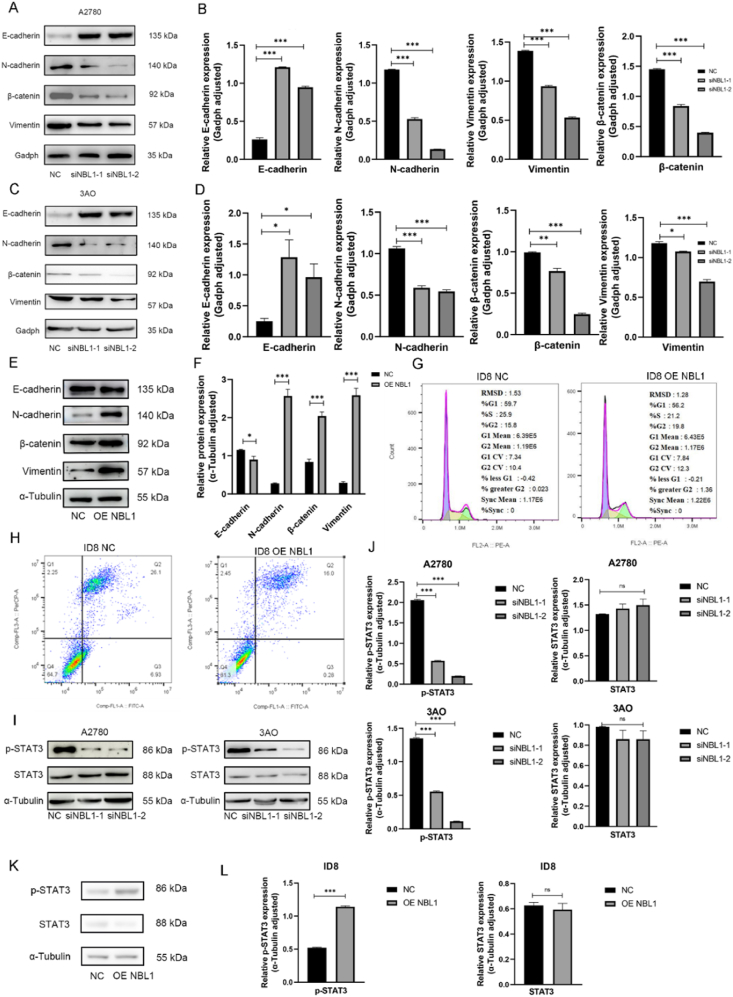
NBL1 enhances the resistance to apoptosis, triggers EMT, and activates the Jak/Stat3 signaling pathway in ovarian cancer. **(A**–**D)** Western blotting analyzed the EMT markers in A2780 cells (A, B) and 3AO cells (C, D). **(E, F)** Western blotting analyzed the EMT markers in ID8 cells. **(G)** The cell cycle was assessed using flow cytometry between ID8 NC and OE NBL1 cells. **(H)** The cell apoptosis was assessed using flow cytometry between ID8 NC and OE NBL1 cells. **(I**–**L)** Western blotting analyzed the Jak/Stat3 signaling pathway markers in A2780, 3AO, and ID8 cells. ∗*p* < 0.05, ∗∗*p* < 0.01, ∗∗∗*p* < 0.001. NBL1, neuroblastoma suppressor of tumorigenicity 1; EMT, epithelial to mesenchymal transition; Jak, Janus kinase; Stat3, signal transducers and activators of transcription 3.

Bioinformatics analysis showed that knocking down NBL1 in OC cells down-regulated E-cadherin and up-regulated N-cadherin, vimentin, and β-catenin (Fig. 6A–D), while elevated NBL1 expression yielded the opposite effect (Fig. 6E, F). Flow cytometry revealed that NBL1 overexpression induced G2/M phase arrest (Fig. 6G). Since actively dividing cells are enriched in G2/M phase, this finding suggests that NBL1 promotes cell cycle progression and proliferation. Besides, cell apoptosis was significantly reduced (Fig. 6H). These findings indicate that NBL1 inhibits cell apoptosis and promotes cancer cell proliferation.

To investigate the impact of NBL1 overexpression on downstream signaling pathways, we analyzed the Jak/Stat3 signaling pathway activation in ID8 cells. NBL1 knockdown reduced phosphorylated Stat3 (p-Stat3) in A2780 and 3AO cells, indicating pathway inhibition (Fig. 6I, J). Conversely, NBL1 overexpression activated the pathway in ID8 cells (Fig. 6K, L), suggesting that NBL1 enhances apoptosis, triggers EMT, and activates the Jak/Stat3 signaling pathway in OC.

### Wp1066, a Stat3 inhibitor, reversed the tumor-promoting effect of NBL1 overexpression

**Figure 7:**
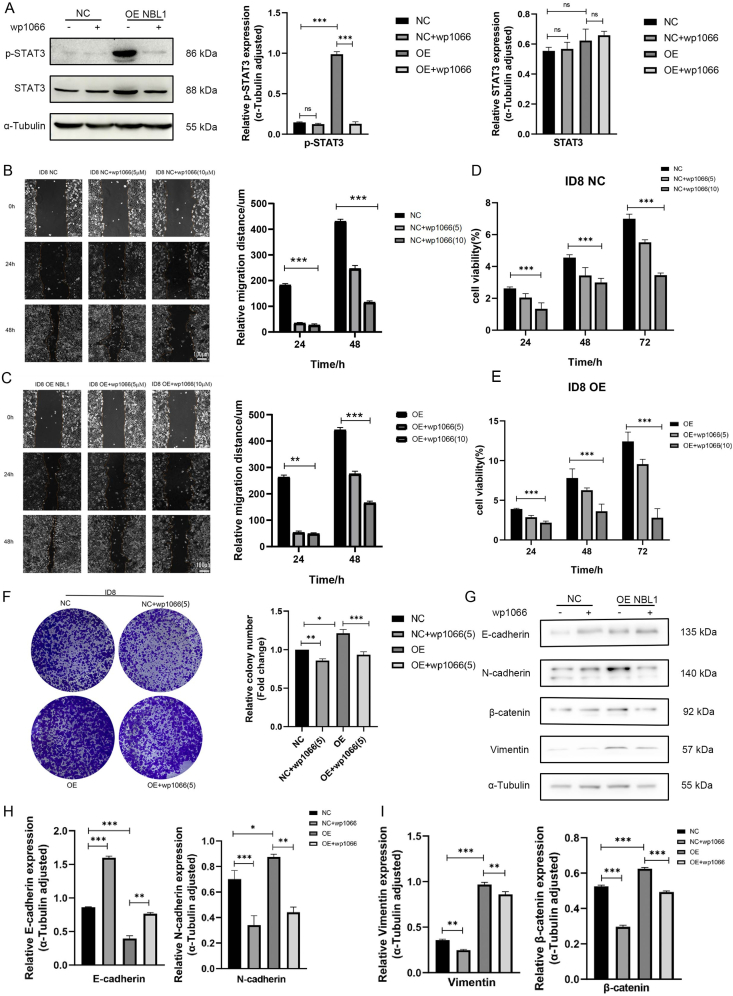
Wp1066 could reverse the tumor-promoting effect of NBL1 overexpression. **(A)** The p-Stat3 and Stat3 protein expression with Wp1066 in ID8 NC and OE NBL1 cells by western blotting analysis. **(B, C)** Wound-healing assay showed the horizontal migration ability in ID8 NC and OE NBL1 cells with Wp1066. **(D, E)** CCK-8 assay showed the effect of Wp1066 on proliferation in ID8 NC and OE NBL1 cells. **(F)** Effects of Wp1066 on colony formation in ID8 NC and OE NBL1 cells. **(G**–**I)** Western blotting showed the effect of Wp1066 on EMT markers in ID8 NC and OE NBL1 cells. ∗*p* < 0.05, ∗∗*p* < 0.01, and ∗∗∗*p* < 0.001. NBL1, neuroblastoma suppressor of tumorigenicity 1; EMT, epithelial to mesenchymal transition; Stat3, signal transducers and activators of transcription 3; p-Stat3, phosphorylated Stat3.

The regulatory role of NBL1 on Stat3 was further confirmed through reverse validation using a Stat3 inhibitor, Wp1066, which blocked the increase in p-Stat3 expression from NBL1 overexpression without affecting total Stat3 levels (Fig. 7A). Wp1066 was introduced into ID8 and overexpressed in cell lines at gradients of 0, 5 μM, and 10 μM. The cell migration distance at both 24 h and 48 h was significantly reduced compared with controls, with the inhibition being concentration-dependent. Wp1066 also negated the migration enhancement from NBL1 overexpression (Fig. 7B, C). The CCK-8 assay and colony formation also demonstrated that Wp1066 reversed the proliferative capacity of NBL1 (Fig. 7D–F). Western blotting analysis revealed that NBL1 overexpression promoted the EMT process, while Wp1066 neutralized this effect (Fig. 7G–I), indicating that the Jak/Stat3 signal pathway mediates NBL1's role in promoting OC.

### The overexpression of NBL1 enhances the metastasis of OC *in vivo*

We established an orthotopic implantation mouse model of OC to investigate the impact of NBL1 on the proliferation and metastasis. Compared with the control group, overexpressing NBL1 showed significantly increased fluorescence intensity, larger ascites volume, and a greater number of metastatic nodules (Fig. S4A). The mean fluorescence intensity of tumors from ID8 cells overexpressing NBL1 in mice was significantly higher than that of the control group (Fig. S4B), with larger ascites volume and weight in the NBL1 overexpression group (Fig. S4C, S4D). Notably, post-sacrifice, 8 mice showed bloody ascites, indicating OC cell invasion and intraperitoneal hemorrhage, promoting tumor spread and metastasis. Consistently, *in vivo* experiments showed that OE NBL1 mice had higher tumor burden (Fig. S4B, S4C), suggesting that NBL1-mediated apoptosis inhibition contributes to aggressive metastasis.

The OE NBL1 group exhibited significantly increased primary tumor burden and extensive metastatic lesions (Fig. S4E). Figure S4F illustrates the initially spread from the contralateral ovary leading to an increased volume and cauliflower-like appearance, with no significant stenosis observed intestinal stasis.

## Discussion

OC is one of the three major gynecological malignancies, characterized by a low early diagnosis rate and poor prognosis. Currently, the main challenge in clinical diagnosis and treatment of OC lies in the lack of sensitive early screening markers and ineffective inhibition of spread and metastasis. Despite advancements in treatment, more than 70% of patients still experience advanced OC spreading, metastasizing, and recurring.^21^ We employed CRISPR gene-editing technology for large-scale genetic screening and established an orthotopic OC mouse model with gene knockout cells.

To mimic tumor metastasis *in vivo*, we used an orthotopic ovarian implantation model in mice, which accurately stimulates the complex interactions of tumor cells and the immune response in the human body, reflecting the origin of OC from the fallopian tube. CRISPR/Cas9 technology enhanced the reliability of target gene screening and validated gene transfer effects both theoretically and practically.

By integrating the GEO dataset, we have pinpointed NBL1 as a pivotal gene influencing the metastasis of OC. The pan-cancer analysis revealed significant variations in NBL1 expression linked to immune cell infiltration and gene mutation. In our research, using six different algorithms, we found a significant negative correlation between NBL1 expression and CD4^+^ T cells as well as CD8^+^ T cells, particularly in OC, indicating that high NBL1 expression suppresses T cell activation and function. CD8^+^ T cells primarily eliminate tumor cells by recognizing specific antigens and class I MHC molecules,^14^^,^^22^ while CD4^+^ T cells activate other immune cells. Nevertheless, the precise mechanism of interaction between NBL1 and immune cells still requires further experimental validation, which is also a limitation of our study.

Contrary to its well-documented tumor-suppressive roles in pancreatic and lung cancers,^14^^,^^23^ our study reveals a paradoxical oncogenic function of NBL1 in OC. In pancreatic ductal adenocarcinoma, NBL1 inhibits tumor growth by antagonizing bone morphogenetic protein 2/4 (BMP2/4) signaling and suppressing EMT, whereas in lung adenocarcinoma, NBL1 acts as a transcriptional target of miR-1301-3p to suppress metastasis. However, in OC, NBL1 overexpression robustly activates Jak/Stat3 signaling and promotes EMT, suggesting a context-dependent duality. This discrepancy may stem from tissue-specific post-translational modifications or microenvironmental crosstalk. For instance, transforming growth factor-beta (TGF-β)/BMP-rich microenvironments in OC could redirect NBL1 from growth suppression (via canonical BMP antagonism) to metastasis promotion through non-canonical Stat3 activation. Additionally, alternative splicing events observed in our RNA-sequencing data (*e.g.*, skipped exons in Fig. S3C) might generate NBL1 isoforms with distinct functional outcomes. Further studies are needed to validate whether splice variants or epigenetic modifications underpin this tissue-specific duality.

The involvement of EMT in cancer metastasis is characterized by the loss of epithelial traits and gain of mesenchymal features, transforming fixed epithelial cells into invasive mesenchymal cells. EMT is linked to various tumor activities^24^ and influenced by various factors, such as inflammatory processes, physical constraints, metabolic stress, and signaling pathways.^23^ Research has indicated that aberrant activation of Wnt, Notch, and TGF-β signaling pathways, along with excessive activation of Ras-extracellular signal-regulated kinase 1/2 (Erk1/2) and nuclear factor kappa B (NF-kB) signaling pathways, can impact the expression of EMT-promoting factors, with NBL1 hypothesized to promote EMT through Wnt and TGF-β signaling. Our study revealed a decrease in E-cadherin expression and an increase in N-cadherin, vimentin, and β-catenin expression following NBL1 overexpression; conversely, when NBL1 was underexpressed, there was an opposite effect (Fig. 6A–F).

Sustained activation of STAT3 is closely associated with various types of tumors, including OC, breast cancer, prostate cancer, and lung cancer.^25^ STAT3 activation influences genes that modulate the survival, proliferation, angiogenesis, invasion, and immune evasion of tumor cells to varying extents.^26^ RNA-sequencing analysis demonstrated that NBL1 overexpression robustly activates Jak/Stat3 signaling (Fig. 5A). While our inhibitor experiments (WP1066) confirmed the functional dependency of NBL1 on Stat3 phosphorylation (Fig. 7A), the direct molecular link remains unclear. We hypothesize that NBL1 may act as a transcriptional co-activator or interact with Stat3 via protein–protein binding to stabilize its active form. This is supported by prior studies showing that TGF-β family members (*e.g.*, BMPs) can modulate Stat3 activity through direct binding. Future experiments, such as co-immunoprecipitation or chromatin immunoprecipitation, are needed to validate these hypotheses.

The strong association between NBL1 overexpression and poor prognosis, coupled with its reversibility by Stat3 inhibitors (*e.g.*, WP1066), positions NBL1 as both a prognostic biomarker and a therapeutic target. Clinically, elevated NBL1 levels in ascites or serum could serve as a non-invasive indicator of metastatic risk, enabling early intervention. Therapeutically, NBL1's dual role (BMP antagonist in pancreatic cancer versus Stat3 activator in OC) highlights the need for tissue-specific strategies. While WP1066 showed efficacy in reversing NBL1-driven metastasis (Fig. 7), its clinical applicability is limited by toxicity. In contrast, FDA-approved Jak/Stat3 inhibitors like ruxolitinib, which target JAK1/2 and improve survival in myelofibrosis, may offer a safer alternative. However, ruxolitinib's efficacy in OC remains unexplored. Future studies should evaluate whether NBL1 expression predicts sensitivity to Jak/Stat3 inhibitors or synergizes with immune checkpoint blockade (*e.g.*, anti-programmed cell death protein 1 or anti-PD1) to overcome T cell exclusion (Fig. S2G, S2H).

Our study has several limitations. First, while we identified NBL1's negative correlation with T cell infiltration via TIMER2.0 and flow cytometry, the direct impact of NBL1 on cancer-associated fibroblasts or myeloid-derived suppressor cells remains unexplored. Given the strong correlation between NBL1 and cancer-associated fibroblast infiltration (Fig. S2D), future work should dissect how NBL1 modulates stromal interactions to foster metastatic niches. Second, although RNA sequencing revealed abundant alternative splicing events (*e.g.*, skipped exons, retained introns) in NBL1-overexpressing cells (Fig. S3C), the functional consequences of these isoforms were not experimentally validated. Third, our inhibitor experiments (WP1066) confirmed Jak/Stat3 dependency but did not explore combinatorial therapies with existing Jak/Stat3-targeted agents (*e.g.*, ruxolitinib). Addressing these gaps will enhance the translational relevance of our findings.

In conclusion, our study uncovers a previously unrecognized oncogenic role of NBL1 in promoting OC metastasis through Jak/Stat3 pathway activation. The strong association between NBL1 overexpression and poor clinical outcomes, coupled with its reversibility by Stat3 inhibitors (*e.g.*, WP1066), positions NBL1 as a potential therapeutic target for metastatic OC. Further validation of its tissue-specific regulatory mechanisms and clinical translation (*e.g.*, antibody-based therapies or Jak/Stat3 inhibitor combinations) is warranted to improve patient survival.

## CRediT authorship contribution statement

**Yue Qi:** Methodology, Conceptualization, Writing – original draft. **Wenwen Zhang:** Writing – review & editing, Visualization. **Xinyu Li:** Data curation, Formal analysis. **Yi Shi:** Conceptualization, Software. **Pengpeng Qu:** Writing – review & editing, Supervision.

## Ethics declaration

The study was conducted in accordance with the Declaration of Helsinki and approved by the Ethics Committee of Tianjin Central Hospital of Gynecology and Obstetrics (approval ID: 2022KY001; date: 25 February 2022). Written informed consent was obtained from all participants or their legal guardians before tissue collection or for publication. For deceased patients, consent was waived by the ethics committee under national regulations.

## Funding

The study was funded by Tianjin Science and Technology Project (No. 24JCYBJC01650) and the Tianjin Education Commission Planning Project (No. 2023YXZD06).

## Conflict of interests

All authors disclosed no conflicting interests.
