# Supplementary material for: A systematic CRISPR screen reveals an NBL1-mediated Jak/Stat3 crosstalk to promote ovarian cancer metastasis
Source: Genes Dis. 2025 Jun 28;13(1):101740. doi: 10.1016/j.gendis.2025.101740 (PMC12557599; doi:10.1016/j.gendis.2025.101740)
Supplement: Multimedia component 1 [file mmc1.docx]

**Supplementary Data**

**Table S1. Correlation between NBL1 expression and clinicopathological characteristics in 381 OC patients in TCGA database.**

| **Characteristics** | **Low expression of NBL1** | **High expression of NBL1** | **P value** |
| --- | --- | --- | --- |
| n | 190 | 191 |  |
| Clinical stage, n (%) |  |  | 0.005 |
| Stage I&Stage II | 19 (5%) | 5 (1.3%) |  |
| Stage III | 147 (38.9%) | 149 (39.4%) |  |
| Stage IV | 23 (6.1%) | 35 (9.3%) |  |
| Tumor status, n (%) |  |  | 0.002 |
| Tumor free | 47 (13.9%) | 25 (7.4%) |  |
| With tumor | 120 (35.5%) | 146 (43.2%) |  |
| Primary therapy outcome, n (%) |  |  | 0.658 |
| PD&SD | 23 (7.4%) | 26 (8.4%) |  |
| PR&CR | 131 (42.4%) | 129 (41.7%) |  |
| Age, n (%) |  |  | 0.873 |
| <= 60 | 105 (27.6%) | 104 (27.3%) |  |
| > 60 | 85 (22.3%) | 87 (22.8%) |  |
| Histologic grade, n (%) |  |  | 0.478 |
| G1 | 0 (0%) | 1 (0.3%) |  |
| G2 | 25 (6.7%) | 20 (5.4%) |  |
| G3&G4 | 164 (44.2%) | 161 (43.4%) |  |
| Venous invasion, n (%) |  |  | 0.008 |
| No | 30 (28.6%) | 11 (10.5%) |  |
| Yes | 30 (28.6%) | 34 (32.4%) |  |
| Lymphatic invasion, n (%) |  |  | 0.137 |
| No | 30 (20.1%) | 18 (12.1%) |  |
| Yes | 50 (33.6%) | 51 (34.2%) |  |
| Anatomic neoplasm subdivision, n (%) |  |  | 0.982 |
| Bilateral | 130 (36.2%) | 127 (35.4%) |  |
| Left | 29 (8.1%) | 27 (7.5%) |  |
| Right | 23 (6.4%) | 23 (6.4%) |  |
| Tumor residual, n (%) |  |  | < 0.001 |
| No | 48 (14.2%) | 20 (5.9%) |  |
| Yes | 120 (35.6%) | 149 (44.2%) |  |
| OS event, n (%) |  |  | 0.024 |
| Alive | 84 (22%) | 63 (16.5%) |  |
| Dead | 106 (27.8%) | 128 (33.6%) |  |


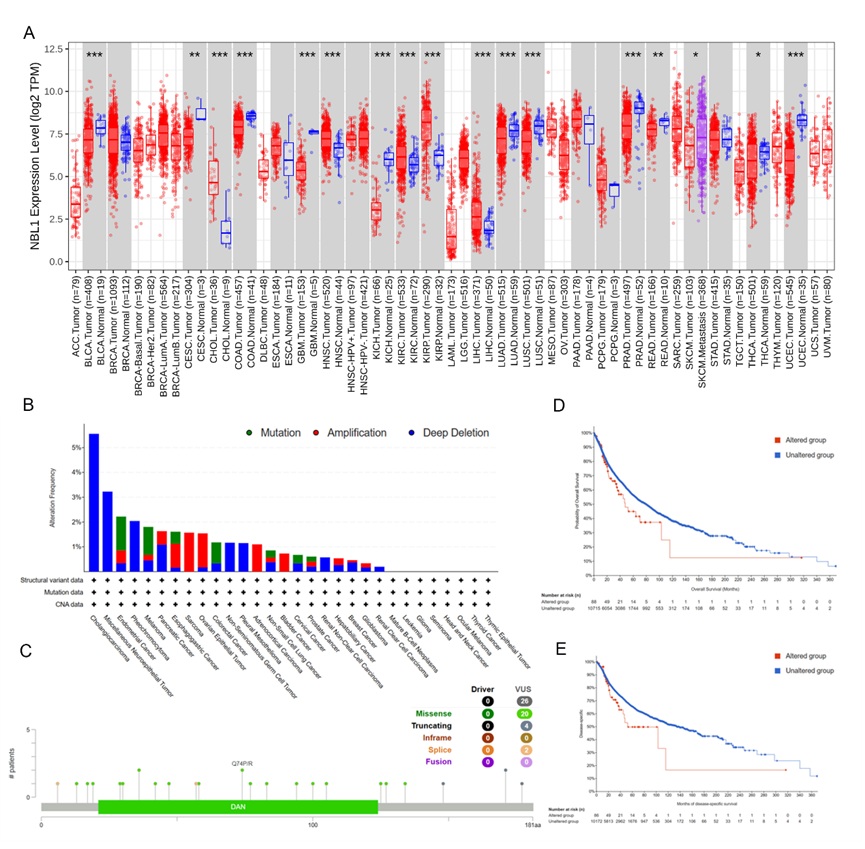


**Figure S1: Pan-cancer analysis of NBL1 expression across various cancer types.** (A): The expression of NBL1 in different tumors in TIMER2.0. Heatmap colors indicate log2-transformed expression levels (red: tumor tissues, blue: normal tissues). Significant differences (adjusted *p<*0.05) are marked with asterisks (*). (B): The different mutation types of NBL1 in different tumors in cBioPortal database and mutation site (C). (D-E): The prognosis relationship between altered group and unaltered group. ( **p<*0.05,***p<*0.01,****p<*0.001)


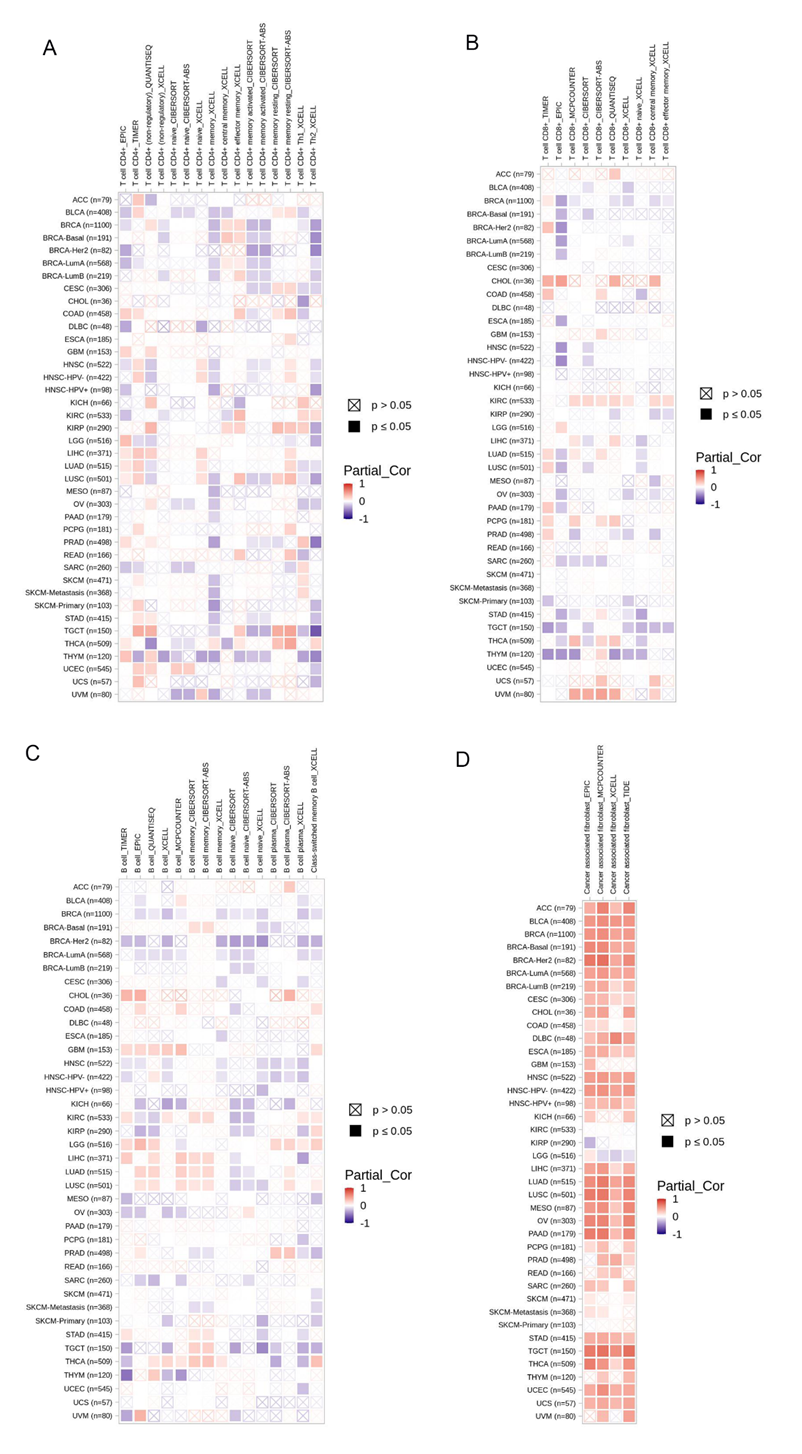


**Figure S2: The relationship between NBL1 expression and immune cells infiltration.** (A): CD4^+^T cells. (B) CD8^+^T cells. (C) B cells. (D) CAF cells. Heatmap colors represent Spearman’s correlation coefficients (red: positive correlation, blue: negative correlation). Cross symbols (☒) indicate non-significant correlations (p≥0.05). TIMER2.0 was used (Version:2.0 and link: <http://timer.cistrome.org/>).


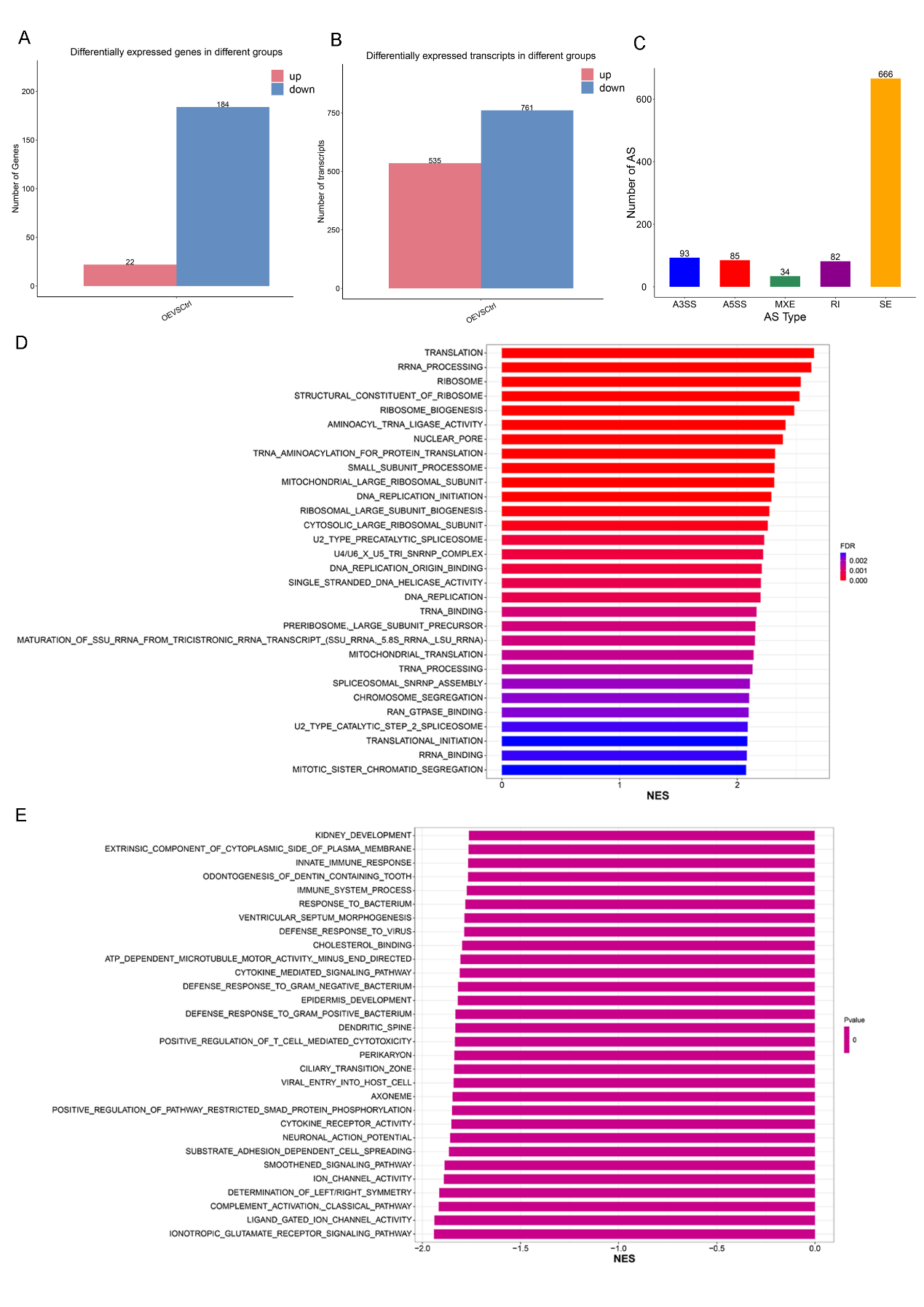


**Figure S3: Potential signal pathway analysis by RNA-seq and online databases.** (A-B): Different expressed genes and transcripts between OE NBL1 and control cells. (C): The changes of alternative splice between OE NBL1 and control cells. (D): The bar graph displays the results of GSEA analysis for the top 30 positively ranked items. (E): The bar graph displays the results of GSEA analysis for the top 30 negatively ranked items.


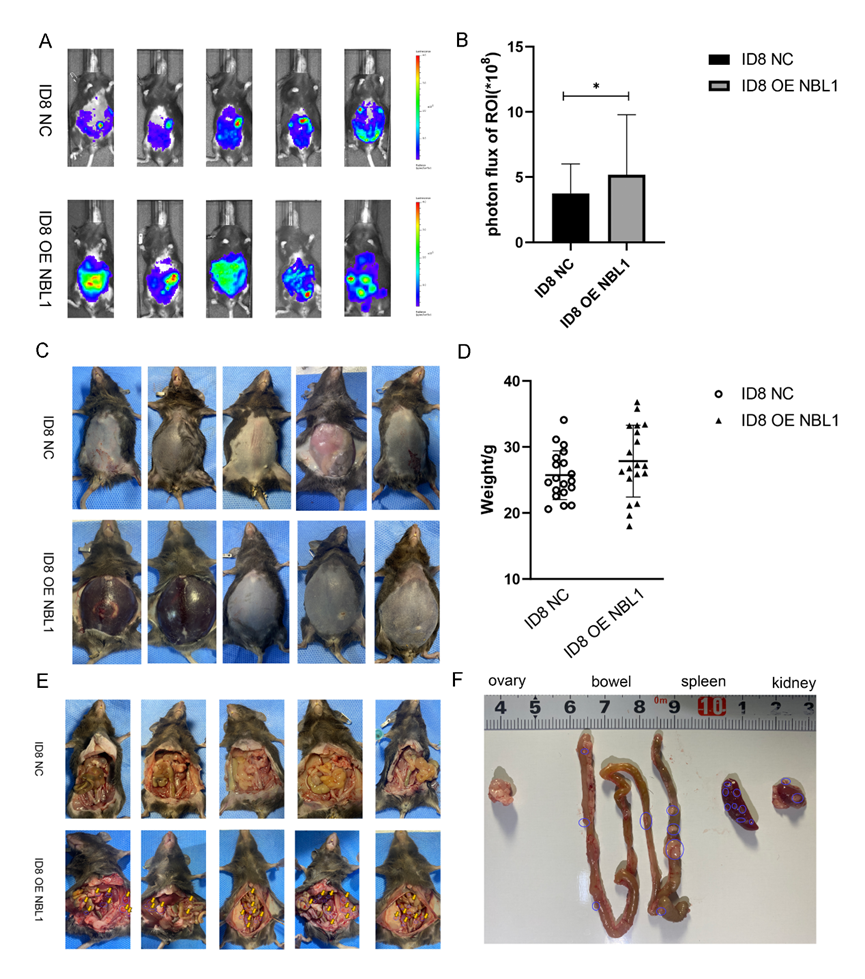


**Figure S4: The NBL1 protein enhances widespread metastasis in mice in vivo of OC.**(A): An orthotopic ovarian cancer model in C57BL/6 mice was established by injecting control ID8 cells or NBL1-overexpressing ID8 cells into left ovary. The fluorescence signal intensity of luciferase-expressing ovarian cancer cells in mice was detected using an in vivo imaging system within 2 months after modeling ([p/s/cm^2^/sr]/[μW/cm^2^]).(B): For fluorescence quantification, regions of interest (ROIs) were delineated using Living Imaging 3.0 software and the total radiation efficiency was calculated (**P<*0.05). (C): Comparison of ascites formation following euthanasia of mice. (D): The body weight of mice in each group was compared (in grams) (**P<*0.05). (E): The development of metastasis in vivo was observed after the sacrifice of mice. (F) Detailed presentation of metastases.
